# Association between maternal sociodemographic characteristics and exclusive mother’s own milk feeding in preterm infants: a cohort study using data from the National Neonatal Research Database

**DOI:** 10.1136/archdischild-2024-327990

**Published:** 2025-02-26

**Authors:** Melissa-Sue Ryan, Lisa Szatkowski, Arrisonia Doubatty, Shalini Ojha

**Affiliations:** 1 Centre for Perinatal Research, Lifespan and Population Health, School of Medicine, University of Nottingham, Nottingham, UK; 2 Neonatal Unit, University Hospitals of Derby and Burton NHS Foundation Trust, Derby, UK

**Keywords:** Neonatology, Epidemiology

## Abstract

**Objective:**

To ascertain the sociodemographic and geographical determinants of exclusive and no mother’s own milk (MOM) feeding for infants <34 weeks’ gestational age (GA) in England and Wales.

**Study design:**

Retrospective cohort study using the National Neonatal Research Database (2016–2022). We calculated unadjusted and mutually adjusted ORs for exclusive and no MOM feeding throughout an infant’s neonatal stay, by maternal age group, ethnicity and deprivation quintile. Neonatal Operational Delivery Network and unit were included as random effects, and the adjusted models included other potential confounders such as gestational age and mode of delivery.

**Results:**

Among the 90 730 infants, 11 962 (13.2%) were exclusively MOM fed, while 9018 (9.9%) never received MOM. The odds of exclusive MOM feeding increased with decreasing maternal social deprivation (OR for least deprived vs most deprived quintile 2.16, 95% CI 2.01 to 2.33), while the odds of no MOM decreased (OR 0.33, 95% CI 0.30 to 0.36). The odds of exclusive MOM feeding were lower for Asian/Asian-British mothers compared with white mothers (OR 0.88, 95% CI 0.82 to 0.95). The odds of never receiving MOM were lower for Black, Asian and mixed ethnicities compared with white mothers. Infants of mothers aged 26–35 years had the highest odds of exclusive MOM feeding. There was a geographical variation in feeding practices with a north-south divide in the prevalence of never receiving MOM. There was a significant variation in feeding practices between neonatal units.

**Conclusion:**

Provision of MOM to preterm infants in England and Wales is associated with maternal sociodemographic characteristics.

**WHAT IS ALREADY KNOWN ON THIS TOPIC**

- The WHO recommends exclusive breastfeeding for at least 6 months for all infants.
- The UK has very low rates of exclusive breastfeeding. Mothers who are younger, of white ethnicity or have higher levels of deprivation have lower rates of breastfeeding.
- Preterm infants gain additional benefits from their mother’s own milk (MOM) feeding as it lowers the risk of necrotising enterocolitis, late-onset infections and neurodevelopmental impairments.
- Rates of exclusive MOM feeding in UK neonatal units are not routinely reported.

**WHAT THIS STUDY ADDS**

- Only 13% of preterm infants in England and Wales are exclusively MOM fed throughout their neonatal stay, and 10% never receive MOM.
- The odds of receiving MOM are associated with the mother’s age, ethnicity and level of deprivation.
- Infants cared for in special care baby units and local neonatal units are less likely to be exclusively MOM fed than infants cared for in neonatal intensive care units.
- There are geographical differences in rates of exclusive MOM feeding and a clear north-south divide and areas of poor outcomes in North Wales and East of England in the percentage of infants who never receive MOM.

**HOW THIS STUDY MIGHT AFFECT RESEARCH, PRACTICE OR POLICY**

- The maternal sociodemographic determinants of MOM feeding are known before preterm birth. Neonatal services should use the opportunity provided by long hospital stays to support and increase the chances of exclusive MOM feeding for every preterm infant, with focus on those at the highest risk of never receiving MOM.
- National quality of care metrics should support this ambition by reporting exclusive, rather than any, MOM feeding, to establish exclusive MOM feeding by at least day 14 and continuing to discharge as the ‘new normal.’
- Research should focus on interventions to improve MOM feeding rates among those who are at the highest risk of not receiving MOM.

## Introduction

 The WHO recommends breastfeeding within an hour of birth and exclusively for 6 months.^1^ In England, 73% infants born in 2022–2023 received breastmilk as their first feed,^2^ but exclusive breastfeeding at 6 months, at around 1%,^3^ is among the lowest worldwide.

Preterm birth is a recognised barrier to breastfeeding.^4^ Infants born before 34 weeks’ gestational age (GA) are unable to breastfeed directly and instead have their mothers’ own milk (MOM) expressed and given via a gastric tube. MOM feeding in preterm infants is associated with multiple additional benefits including lower incidences of necrotising enterocolitis (NEC), late-onset infections, bronchopulmonary dysplasia, retinopathy of prematurity and neurodevelopmental impairments.^4^ Where MOM is unavailable, the WHO recommends that preterm infants should receive donor human milk (DHM).^5^

The UK National Neonatal Audit Programme assesses whether neonatal services provide high-quality care. It includes a composite metric which includes milk fed on day 14 and at discharge home.^6^ This measures ‘any’ MOM and does not report exclusive MOM feeding throughout the entirety of the neonatal stay. Currently, there are no reports of the extent of exclusive MOM feeding among preterm infants in the UK and no studies have evaluated its determinants. Similarly, there is no recognition of those who never receive MOM. Without these data, it is difficult for UK neonatal services and families of preterm infants to determine how and where to invest to promote MOM feeding.

We aimed to determine the prevalence of exclusive and no MOM feeding among preterm infants in England and Wales and to describe the maternal sociodemographic and neonatal service characteristics associated with exclusive MOM feeding and never receiving MOM. Families were involved in prioritising the research questions.^7^

## Methods

We performed a retrospective cohort analysis using data from the National Neonatal Research Database (NNRD), as previously described,^8^ for infants born at 22–33 weeks’ GA in England and Wales from 1 January 2016 to 31 December 2022.

### Exclusions

We excluded infants who had missing data on sex or birth weight; had an implausible birth weight for gestational age z-score (>4 SD or <-4 SD, calculated using the *zanthro* function in Stata with reference to the UK-WHO preterm growth standards^9^; were admitted >24 hours after birth; were cared for in more than one hospital and records were not available from one or more of these episodes; were born with a fatal congenital anomaly (online supplemental table 1); and who were never fed enterally.

### Outcomes

We defined exclusive MOM as only MOM feeding throughout neonatal stay, and no MOM as no record ever of MOM feeding. Exclusive and no human milk (MOM and/or DHM) feeding were similarly defined. We identified whether infants received any MOM during the first 2 days of care and the type(s) of feed on day 14 and the final day of care (the day of death or discharge). Exclusive formula feeding was defined as receiving only formula milk; mixed feeding included any combination of formula, MOM and/or DHM.

### Exploratory variables

Maternal age was recorded in completed years and grouped into: <16, 16–25, 26–35, 36–45, 46+. Maternal ethnicity was grouped according to the UK Office for National Statistics categories: White, Mixed, Asian/Asian-British, Black/Black-British, Other. Maternal deprivation was categorised using the Index of Multiple Deprivation (IMD)^10^ quintile of the home postcode. Postcodes were also mapped to 2021 Local Authority Districts (LAD) to indicate geographical location.

Maternal age, ethnicity, IMD and LAD of residence are markers of maternal sociodemographic status. Data from community settings demonstrate the importance of sociodemographic indicators of breastfeeding,^11^ although these have not been studied in the preterm population where breastfeeding is established during in-hospital care.

### Other confounders

We identified a number of other variables that may determine feeding practices and outcomes, including mode of delivery (vaginal vs caesarean section); infants’ clinical condition at birth (operationalised using the NMR-2000 score^12^ for risk of in-hospital mortality); whether an infant was transferred between units in the first 48 hours after birth; and total length of hospital stay. Neonatal units in the UK are grouped geographically into Operational Delivery Networks (ODNs).^13^ Units within an ODN work together to deliver care at different levels of intensity^14^ (level 3, neonatal intensive care units (NICUs); level 2, local neonatal units (LNUs); and level 1, special care baby units (SCBUs)). Infants are often transferred between units in an ODN for clinical or capacity reasons, and knowledge, skills and practices are shared between units. We identified the unit and ODN in which infants were being cared for on day 3. For infants with a length of stay <2 days, we used the location of care on day 1.

### Statistical analysis

Data management and analysis were performed using Stata V.18 (StataCorp, College Station, Texas, USA) and R and RStudio.^15 16^ NNRD has complete coverage of all admissions to NHS neonatal units in England and Wales.^17^ All births below 34 weeks’ GA are admitted for neonatal care. We used all available data, without sampling.

After exclusions, we described the characteristics of infants and mothers, overall and by GA group (most preterm, 22–24 weeks’ GA; extremely preterm, 25–27; very preterm, 28–31; moderately preterm 32–33). We quantified the proportion of infants who received exclusive MOM and no MOM, and exclusive and no human milk, throughout care, and described their sociodemographic and clinical characteristics. We also quantified feeding type on day 14 and the final day of care, and the day each milk type was first received, by ODN, year of birth and GA group.

We calculated unadjusted OR for exclusive MOM and no MOM, and exclusive and no human milk, by maternal age group, ethnicity and IMD quintile. We then calculated adjusted ORs, first mutually adjusting for age, ethnicity and deprivation, and then with further adjustment for characteristics measured at the level of the infant (GA in completed weeks; sex; multiple birth; mode of delivery; categorised NMR-2000 score indicating risk of in-hospital mortality^12^; inter-hospital transfer in first 48 hours of life; length of hospital stay (in 2-week bands) and the unit (BAPM unit level). To account for clustering, our adjusted models included random effects for ODN and unit.

We mapped the percentage of infants who received exclusive and no MOM, and exclusive and no human milk, by LAD. We used stacked bar charts to explore variations in the crude percentage of infants who received exclusive and no MOM and human milk by combinations of IMD quintile and ethnicity, IMD and maternal age, and maternal age and ethnicity.

## Results

From 93 733 infants, 90 730 were included (see online supplemental figure 1 for exclusions). Baseline characteristics are in the online supplemental table 2.

**Figure 1:**
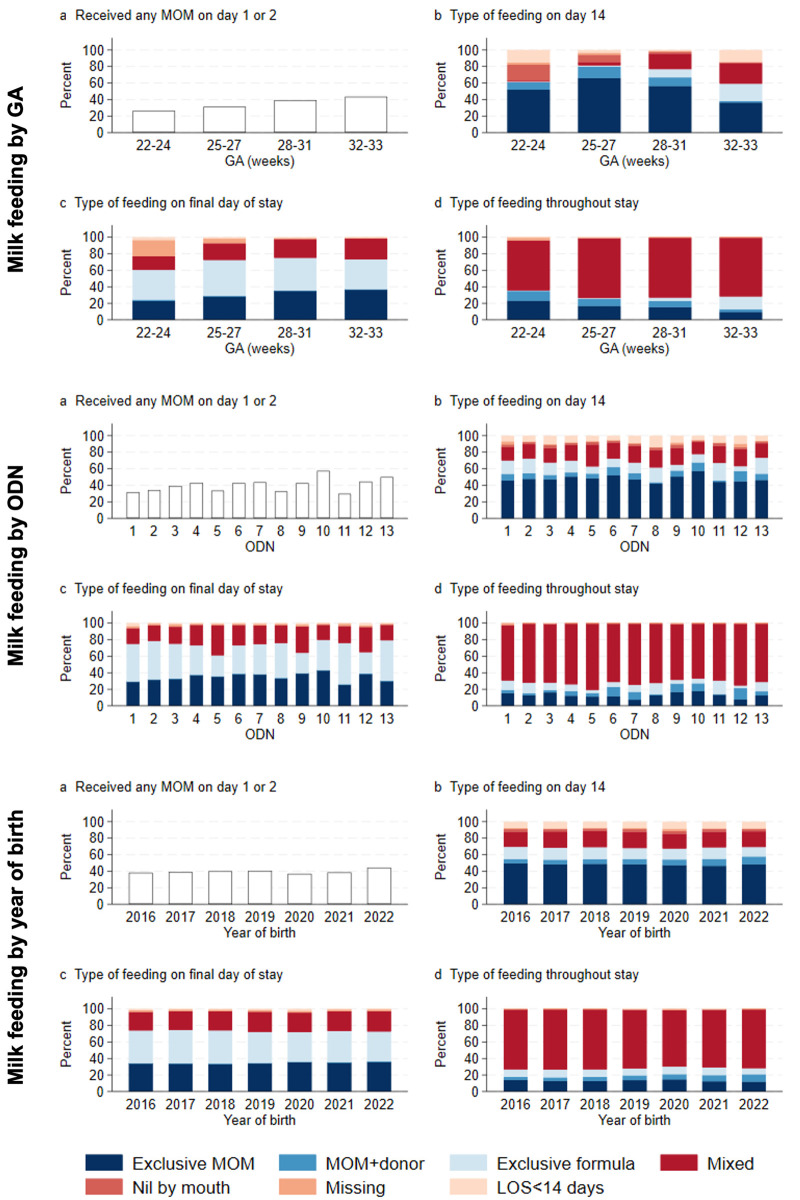
Early mother’s own milk feeding (MOM) (**a**), milk fed on day 14 (**b**), milk fed on the last day of neonatal care (**c**) and milk fed throughout neonatal care (**d**) by gestational age (GA) (top), neonatal operational delivery network (ODN) (middle) and year of birth (bottom). LOS, length of stay.

Figure 1 and online supplemental table 3 show early milk feeding and the type of milk on day 14, the last day of neonatal care and throughout care, by GA group, ODN, and year of birth. Overall, 36 021 (39.7%) infants received some MOM in the first 2 days; the median (IQR) day of first MOM feed was 3 (2–4) days. Prevalence of exclusive MOM feeding on day 14 was 55.0% and reduced to 34.7% by discharge. The most and extremely preterm infants had the highest rate of exclusive MOM feeding at day 14 but the lowest rates at discharge. Rates of these outcomes did not change over the study period. Overall, 11 962 (13.2%) infants were exclusively MOM fed throughout care and 9018 (9.9%) never received MOM. A total of 17 261 (19.0%) were exclusively human milk fed, while 8033 (8.9%) never received human milk.

Online supplemental table 4 shows the characteristics of infants who were exclusively MOM fed, who never received MOM and those who were mix fed. Infants who were exclusively MOM fed were born at a lower GA and more were male compared with those who never received MOM.

### Variations by maternal sociodemographic characteristics

**Figure 2:**
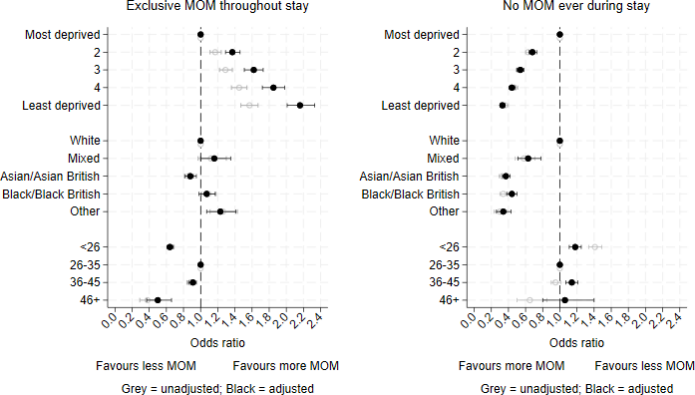
Adjusted (black) and unadjusted (grey) ORs (95% CIs) for exclusively receiving their mother’s own milk (MOM) and not receiving any MOM throughout neonatal care in infants <34 weeks’ gestational age admitted for neonatal care in England and Wales (2016–2022).

Figure 2 and table 1 show the adjusted and unadjusted ORs for exclusive MOM feeding throughout care, and never receiving MOM, by maternal IMD, ethnicity and age category. Similar figures for exclusive and no human milk are in online supplemental figure 2 and table 5.

**Table 1:** Unadjusted and adjusted ORs for exclusive and no MOM, by maternal sociodemographic characteristics

		Exclusive MOM throughout stay			No MOM ever during the stay		
		Unadjusted	Mutually adjusted+clustered	Fully adjusted+clustered	Unadjusted	Mutually adjusted+clustered	Fully adjusted+clustered
IMD quintile	Most deprived	Ref	Ref	Ref	Ref	Ref	Ref
	2	1.17 (1.11–1.24)	1.34 (1.26–1.43)	1.37 (1.29–1.46)	0.63 (0.60–0.67)	0.69 (0.65–0.74)	0.68 (0.64–0.73)
	3	1.29 (1.22–1.37)	1.55 (1.46–1.66)	1.62 (1.51–1.73)	0.52 (0.49–0.56)	0.55 (0.51–0.59)	0.54 (0.50–0.58)
	4	1.45 (1.36–1.54)	1.73 (1.62–1.85)	1.85 (1.72–1.98)	0.47 (0.44–0.51)	0.47 (0.43–0.51)	0.44 (0.41–0.48)
	Least deprived	1.57 (1.47–1.67)	1.99 (1.85–2.14)	2.16 (2.01–2.33)	0.36 (0.33–0.40)	0.36 (0.32–0.39)	0.33 (0.30–0.36)
	Missing	1.22 (1.13–1.33)	1.37 (1.21–1.56)	1.44 (1.26–1.64)	0.70 (0.64–0.76)	0.77 (0.67–0.88)	0.72 (0.62–0.82)
Maternal ethnic group	White	Ref	Ref	Ref	Ref	Ref	Ref
	Mixed	1.12 (0.97–1.30)	1.16 (1.00–1.35)	1.16 (1.00–1.35)	0.58 (0.48–0.71)	0.63 (0.52–0.78)	0.63 (0.51–0.78)
	Asian/Asian British	0.86 (0.81–0.92)	0.94 (0.88–1.01)	0.88 (0.82–0.95)	0.33 (0.29–0.36)	0.36 (0.32–0.40)	0.37 (0.33–0.42)
	Black/Black British	1.09 (1.01–1.18)	1.19 (1.09–1.29)	1.07 (0.98–1.17)	0.34 (0.30–0.39)	0.39 (0.34–0.44)	0.44 (0.38–0.50)
	Other	1.26 (1.11–1.43)	1.28 (1.12–1.46)	1.23 (1.07–1.41)	0.30 (0.23–0.38)	0.34 (0.27–0.44)	0.34 (0.26–0.43)
	Missing	0.96 (0.92–1.01)	0.99 (0.94–1.05)	0.96 (0.91–1.02)	0.89 (0.85–0.94)	0.91 (0.86–0.96)	0.93 (0.87–0.99)
Maternal age group (years)	<26	0.65 (0.62–0.69)	0.68 (0.64–0.72)	0.64 (0.61–0.68)	1.41 (1.34–1.49)	1.13 (1.07–1.20)	1.18 (1.11–1.25)
	26–35	Ref	Ref	Ref	Ref	Ref	Ref
	36–45	0.88 (0.84–0.93)	0.87 (0.83–0.91)	0.91 (0.86–0.95)	0.95 (0.90–1.01)	1.12 (1.06–1.19)	1.14 (1.07–1.21)
	46+	0.38 (0.29–0.50)	0.38 (0.29–0.50)	0.50 (0.37–0.66)	0.65 (0.50–0.85)	1.02 (0.78–1.34)	1.06 (0.80–1.40)
	Missing	0.84 (0.68–1.03)	0.88 (0.70–1.09)	0.90 (0.72–1.12)	1.41 (1.15–1.74)	1.26 (1.01–1.56)	1.14 (0.91–1.42)
Gestational age (weeks)	33			Ref			Ref
	32			1.46 (1.36–1.57)			0.83 (0.78–0.88)
	31			1.93 (1.77–2.11)			0.66 (0.60–0.72)
	30			2.28 (2.07–2.52)			0.58 (0.52–0.66)
	29			3.40 (3.05–3.80)			0.42 (0.36–0.49)
	28			4.33 (3.85–4.87)			0.30 (0.25–0.37)
	27			5.26 (4.62–5.99)			0.27 (0.22–0.35)
	26			5.61 (4.90–6.42)			0.17 (0.13–0.23)
	25			5.12 (4.43–5.92)			0.13 (0.09–0.20)
	24			5.32 (4.57–6.21)			0.19 (0.13–0.26)
	23			6.78 (5.64–8.15)			0.20 (0.12–0.31)
	22			7.39 (5.21–10.49)			0.39 (0.19–0.77)
Sex	Male			Ref			Ref
	Female			0.91 (0.87–0.94)			1.05 (1.01–1.10)
Multiple birth	Singleton			Ref			Ref
	Multiple			0.55 (0.52–0.57)			1.05 (0.99–1.11)
Mode of delivery	Vaginal			Ref			Ref
	Caesarean			0.95 (0.91–1.00)			0.89 (0.85–0.94)
	Missing			0.88 (0.80–0.97)			0.98 (0.89–1.08)
Acute post-natal transfer	No			Ref			Ref
	Yes			0.94 (0.87–1.00)			1.06 (0.97–1.16)
NMR-2000 risk of in-hospital mortality	Low risk			Ref			Ref
	Medium risk			1.40 (1.31–1.50)			0.83 (0.77–0.89)
	High risk			1.48 (1.30–1.68)			0.71 (0.52–0.96)
	BW >2000 g			0.97 (0.90–1.04)			1.07 (1.01–1.14)
	Missing			1.20 (1.09–1.32)			0.98 (0.88–1.08)
Length of stay (weeks)	>8			Ref			Ref
	6–8			2.65 (2.45–2.87)			1.19 (1.04–1.37)
	4–6			3.45 (3.17–3.74)			1.33 (1.17–1.52)
	2–4			3.91 (3.57–4.27)			2.18 (1.91–2.48)
	≤2			5.35 (4.83–5.92)			3.77 (3.27–4.34)
Unit level	Level 3 (NICU)			Ref			Ref
	Level 2 (LNU)			0.75 (0.61–0.92)			1.03 (0.90–1.18)
	Level 1 (SCBU)			0.55 (0.42–0.71)			0.96 (0.82–1.14)
	Missing			0.81 (0.44–1.49)			0.91 (0.45–1.84)
LR test χ^2^ vs logistic model (p value)			2620.440 (<0.001)	2376.081 (<0.001)		1509.273 (<0.001)	829.085 (<0.001)
Variance (SE) of random intercept for ODN			0.045 (0.031)	0.060 (0.036)		0.113 (0.051)	0.103 (0.044)
Variance (SE) of random intercept for unit			0.358 (0.047)	0.300 (0.040)		0.211 (0.028)	0.107 (0.016)

BW, birth weight; IMD, Index of Multiple Deprivation; LNU, local neonatal unit; MOM, mother’s own milk; NICU, neonatal intensive care unit; ODN, Operational Delivery Network; SCBU, special care baby unit.

The odds of exclusive MOM feeding increased with decreasing maternal deprivation (OR for least deprived vs most deprived quintile 2.16, 95% CI 2.01 to 2.33), while the odds of no MOM decreased (OR 0.33, 95% CI 0.30 to 0.36). Compared with infants of white mothers, the odds of exclusive MOM feeding were lower for infants of Asian/Asian-British mothers (OR 0.88, 95% CI 0.82 to 0.95) and higher for infants of mothers of mixed (OR 1.16, 95% CI 1.00 to 1.35) and other ethnicities (OR 1.23, 95% CI 1.07 to 1.41). The odds of never receiving any MOM were lower for Black, Asian and mixed groups compared with infants of white mothers. Infants of younger and older mothers had lower odds of exclusive MOM feeding and higher odds of never receiving any MOM compared with infants of mothers aged 26–35 years.

**Figure 3:**
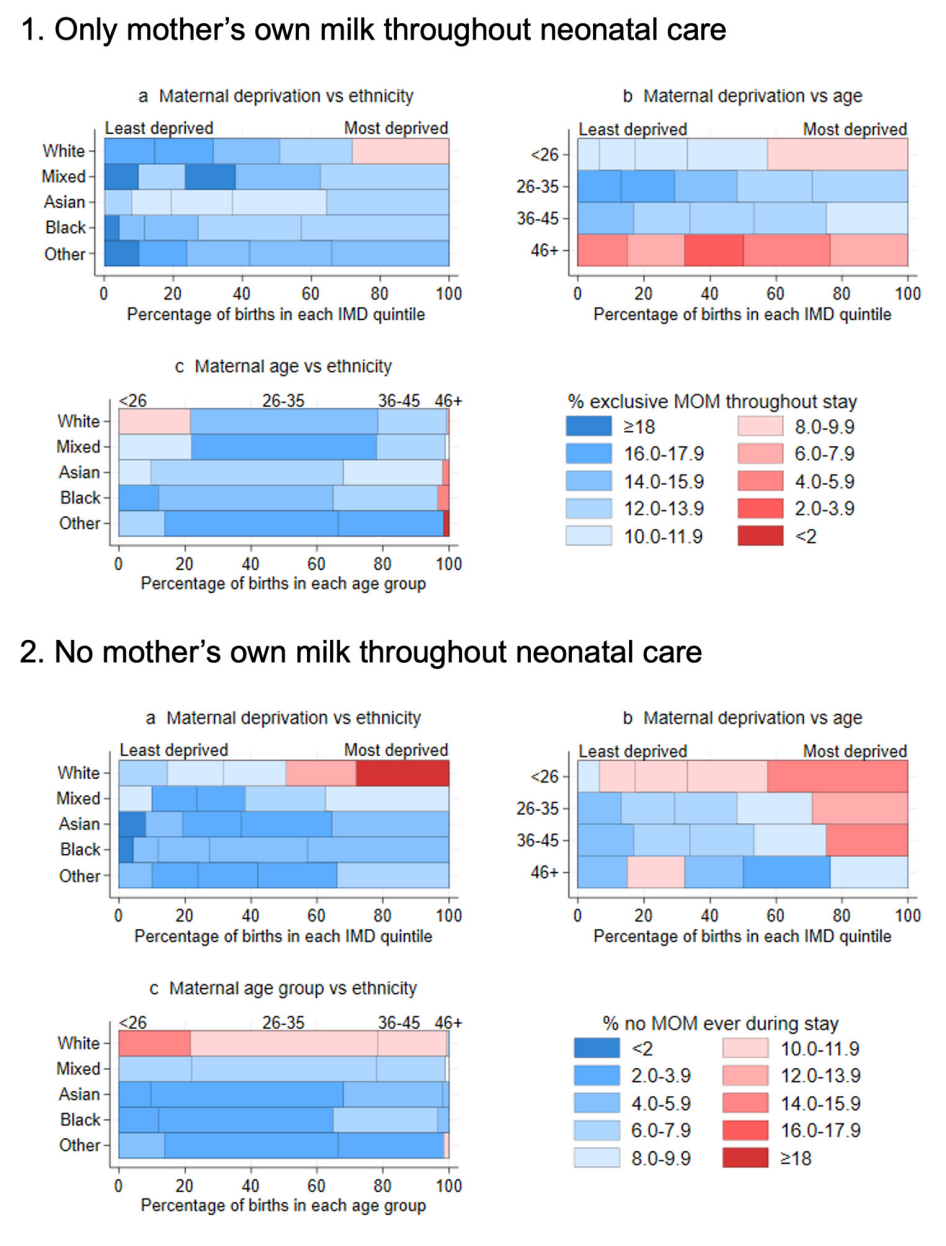
Exclusive mother’s own milk (MOM) feeding (1) and no MOM feeding (2) throughout neonatal care by maternal Index of Multiple Deprivation (IMD) quintile and ethnicity (**a**), IMD and age (**b**), and age and ethnicity (**c**). Data suppressed for groups with <20 infants.

Figure 3 shows the intersection in variations in exclusive MOM feeding by maternal deprivation versus ethnicity, maternal deprivation versus age, and maternal age versus ethnicity. The lowest prevalence of exclusive MOM feeding was seen in the infants of mothers who are <26 years old, white and most deprived. Infants of the most deprived mothers, regardless of their age, and the most deprived white mothers had the highest prevalence of no MOM feeding. Data for exclusive human milk feeding are shown in online supplemental figure 3 and for variations by disaggregated ethnic group in online supplemental figure 4.

### Geographical variations by LAD

**Figure 4:**
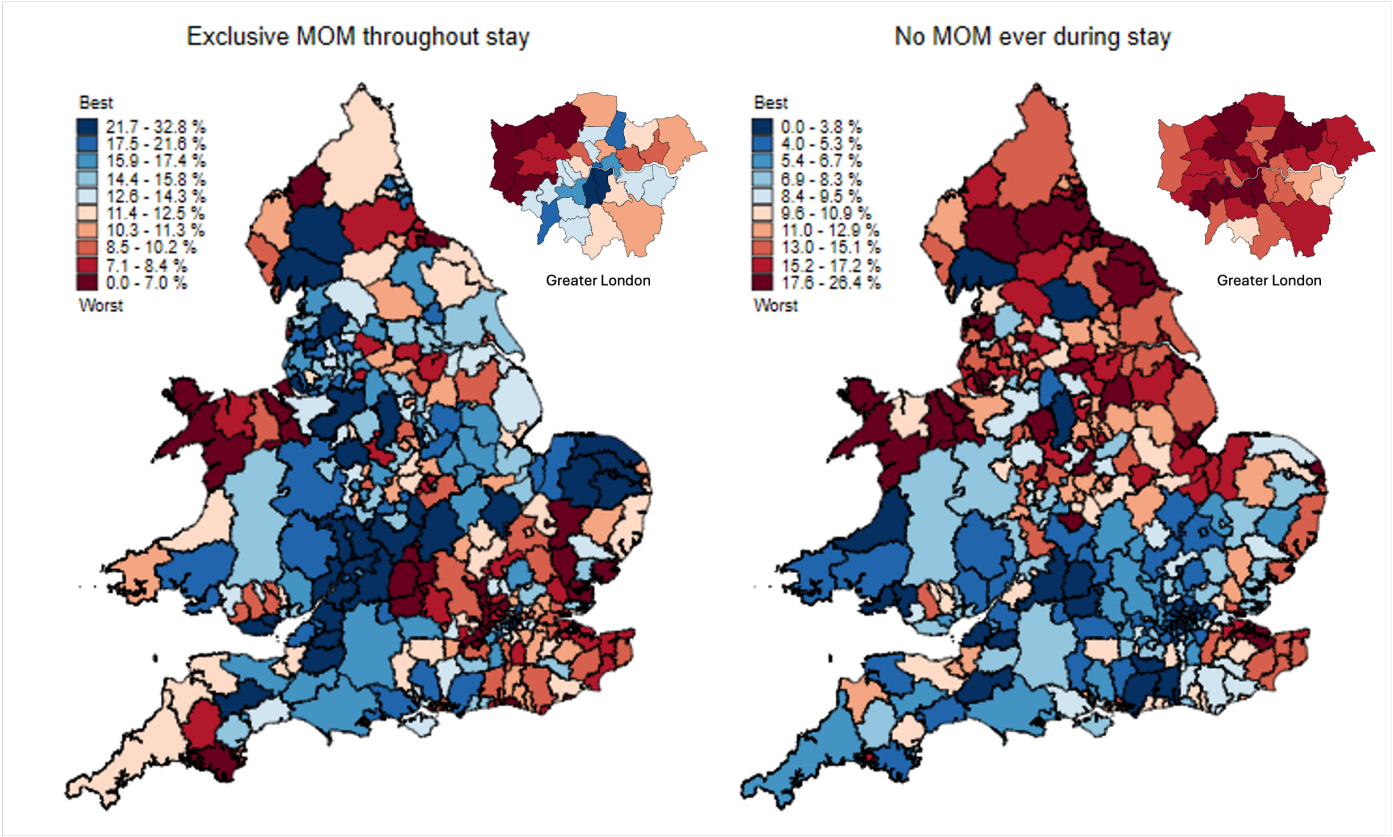
Percentage of infants by maternal Local Authority District who exclusively received their mother’s own milk (MOM) and did not receive any MOM throughout neonatal care in infants <34 weeks’ gestational age admitted for neonatal care in England and Wales (2016–2022).

Figure 4 shows the percentage of infants by maternal LAD who received exclusive MOM and no MOM throughout care. Human milk feeding is shown in online supplemental figure 5. The prevalence of exclusive MOM feeding by LAD ranged from 0.0% to 32.8%, but there was no clear geographical pattern in prevalence. The prevalence of no MOM feeding ranged from 0.0% to 26.4%, with the LADs with the highest prevalence being concentrated in the north and midlands of England, north Wales, north and east Kent, and some areas of Greater London.

### Variations by neonatal care provider

There was substantial variation in the crude prevalence of exclusive MOM feeding between ODNs (range 7.4% to 17.7%) as well as the prevalence of receiving no MOM (range 4.2% to 16.5%). In our fully adjusted multilevel models, there remained significant variation between ODNs in the prevalence of no MOM feeding (variance of random intercept 0.103, SE 0.044) but no significant variation in exclusive MOM feeding (online supplemental table). Infants cared for in level 2 (LNU) and level 1 (SCBU) units had lower odds of exclusive MOM and human milk feeding compared with infants cared for in level 3 NICUs. There was a significant variation in both exclusive and no MOM and human milk feeding between units.

## Discussion

Only 13% of preterm infants who receive neonatal care in the UK are exclusively MOM fed throughout their stay, and less than a fifth meet the WHO recommendation of feeding exclusively human milk.^1 2^ The odds of exclusive MOM feeding and of never receiving MOM are associated with maternal sociodemographic factors. After adjustment for potential confounders, there remains a significant variation in feeding outcomes between neonatal units.

UK surveys including infants of all gestations report low breastfeeding rates with persistent inequalities. Older women with higher levels of education, or from ethnic minorities, are more likely to breastfeed.^18^ We found that preterm infants born to mothers aged 26–35 years were more likely to be exclusively MOM fed and less likely to never receive MOM compared with infants born to older or younger mothers. Lower breastfeeding rates among younger women are attributed to older, more educated mothers being more likely to seek or encounter breastfeeding information.^18^ This difference should not apply to mothers who deliver preterm who establish feeding while their infants are in hospital, often for weeks to months, with adequate opportunity for services to provide information and support.

We found that infants of mothers of Black, Asian, Mixed or other ethnicities were less likely to never receive MOM than infants of white mothers. The 2020–2021 NHS England data show that, at 6–8 weeks, white mothers had the lowest rate of any breastfeeding and the highest rate of no breastfeeding.^19^ However, contrary to the national rates, we found that preterm infants of Asian/Asian-British mothers were less likely to receive exclusive MOM compared with infants of white mothers. Previous UK studies suggest that ethnically dense communities can embody the normality of breastfeeding, providing those living within them with the ‘minority ethnic advantage’.^18^ Such advantages may not carry through when an infant is in hospital, the mother is isolated from her community, and professional services do not meet her needs. A qualitative study among British-Bangladeshi women showed that provision of breastfeeding support was both inappropriate and inaccessible, and practitioners’ assumptions and cultural stereotypes result in unmet needs.^20^

We also found that exclusive MOM feeding increased, and no MOM feeding decreased, with decreasing maternal deprivation. Infants cared for in neonatal units designated for lower levels of care had worse rates of MOM feeding compared with those in level 3 NICUs. There was a significant variation between ODNs and units in the odds of no MOM feeding, and between units in the odds of exclusive MOM feeding. Further, we mapped the rates of exclusive and no MOM feeding by LAD demonstrating stark regional inequalities. We expected that the disadvantages imposed by maternal sociodemographic characteristics would be, at least partially, mitigated by support for breastfeeding while in neonatal care. However, results demonstrate that current services fail to address this.

The British Association of Perinatal Medicine recommends optimisation of early MOM, which includes first MOM feed ‘ideally within 6 hours and always within 24 hours’.^21^ We found that only 40% of infants receive any MOM by day 2. The benefits of early MOM feeding are sustained only if mothers are supported to continue to express.^22^ Valentine *et al* showed that for every 1% increase in MOM feeds in the first 14 and 28 days, the odds of discharge home on exclusive MOM feeding increased by 7- and 17-fold, respectively.^23^ Preterm birth comes with several factors that inhibit the natural establishment of breastfeeding, including enhanced anxiety and mother-infant separation.^23^ Success of breastfeeding is predicated on sustained professional support.

Previous studies in other similar settings have shown that infants born at lower GAs are less likely to be MOM fed at discharge,^24 25^ attributing this to infants’ immaturity and the complications of extreme prematurity.^24^ We found that exclusive MOM feeding was initially very high in the more immature infants. Over three-quarters of infants <28 weeks’ GA were exclusively MOM fed on day 14. In these early days, there is a high risk of NEC and infections. MOM feeding is promoted robustly because it is protective, but it appears that support is not sustained. By discharge, exclusive MOM feeding reduced to 30% and nearly half were on no MOM; infants <28 weeks’ GA had lower rates of MOM feeding, and higher rates of no MOM feeding, compared with more mature infants.

We present data from nearly all infants <34 weeks’ GA over a recent 7-year period in England and Wales. Only small numbers were excluded due to missing or poor-quality data. The majority of exclusions were infants who were never enterally fed, representing the most unwell infants who die soon after birth; their exclusion does not affect the generalisability of our results. A small amount of daily feeding data was missing, and we acknowledge that data quality is reliant on clinical staff in the contributing units.

We did not have data to explore other factors such as maternal education level and father’s (or other parent’s) characteristics as these are not well recorded in the NNRD. We also did not consider the many potential interactions between characteristics in our modelling, for example, we were not able to investigate the effects of service-level differences such as UNICEF Baby Friendly accreditation^26^ and DHM availability. Strategies including skin-to-skin contact, peer support, use of mechanical pumps, multidisciplinary training and Baby Friendly accreditation are evidence-based, cost-effective interventions that promote MOM feeding in neonatal units.^27^ We were unable to test these as the NNRD does not include the required data.

Our analysis focuses on exclusive MOM and human milk feeding. The UK National Neonatal Audit Programme and other studies report point prevalence of ‘any’ breastfeeding without adjustments for infant or maternal characteristics. These do not represent the true extent of the inequalities. There are several non-modifiable infant and maternal characteristics, known at or before birth, that are strongly linked to MOM feeding and the risk of never receiving MOM. This should provide an opportunity to focus support on those with the highest risk such that inequalities are reduced. While it may be difficult to achieve exclusive MOM feeding throughout neonatal care, such as in mothers who are unwell or otherwise unable to provide sufficient MOM in the first few days, exclusive MOM feeding should be possible by day 14 and thereafter until discharge. To highlight this, the national quality of care metrics such as those in the National Neonatal Audit Programme^6^ could be more ambitious and report exclusive MOM feeding. Sustained breastfeeding support and the integration of MOM feeding promotion into daily workflow, with ongoing data-driven feedback,^4^ could establish a new normal^1^ where every preterm infant can expect to be exclusively MOM fed and every mother can have the support to enable it.

## Supplementary material

10.1136/archdischild-2024-327990 online supplemental file 1

## Data Availability

Data are available upon reasonable request. Data may be obtained from a third party and are not publicly available.

## References

[1] The Lancet (2016). Breastfeeding: achieving the new normal. The Lancet.

[2] NHS Digital (2022). NHS maternity statistics, england, 2022-23. https://digital.nhs.uk/data-and-information/publications/statistical/nhs-maternity-statistics/2022-23/births#breast-milk-as-first-feed.

[3] McAndrew F, Thompson J, Fellows L (2012). Infant feeding survey 2010. https://files.digital.nhs.uk/publicationimport/pub08xxx/pub08694/infant-feeding-survey-2010-consolidated-report.pdf.

[4] Parker MG, Stellwagen LM, Noble L (2021). Promoting Human Milk and Breastfeeding for the Very Low Birth Weight Infant. Pediatrics.

[5] Quigley M, Embleton ND, McGuire W (2019). Formula versus donor breast milk for feeding preterm or low birth weight infants. Cochrane Database Syst Rev.

[6] National Neonatal Audit Programme (2024). A guide to the 2024 audit measures. https://www.rcpch.ac.uk/sites/default/files/2024-01/2024_nnap_audit_measures_guide_v1.0_0.pdf.

[7] Abramson J, Szatkowski L, Bains M (2024). Effects of implementation of a care bundle on rates of necrotising enterocolitis and own mother’s milk feeding in the East Midlands: protocol for a mixed methods impact and process evaluation study. BMJ Open.

[8] Szatkowski L, Sharkey D, Budge H Association between opioid use during mechanical ventilation in preterm infants andevidence of brain injury: a propensity-score matched cohort study. https://papers.ssrn.com/sol3/papers.cfm?abstract_id=4478247.

[9] Vidmar SI, Cole TJ, Pan H (2013). Standardizing Anthropometric Measures in Children and Adolescents with Functions for Egen: Update. The Stata Journal: Promoting Communications on Statistics and Stata.

[10] Ministry of Housing, Communities & Local Government English indices of deprivation 2019. https://www.gov.uk/government/statistics/english-indices-of-deprivation-2019.

[11] Oakley LL, Renfrew MJ, Kurinczuk JJ (2013). Factors associated with breastfeeding in England: an analysis by primary care trust. BMJ Open.

[12] Medvedev MM, Brotherton H, Gai A (2020). Development and validation of a simplified score to predict neonatal mortality risk among neonates weighing 2000 g or less (NMR-2000): an analysis using data from the UK and The Gambia. Lancet Child Adolesc Health.

[13] British Association of Perinatal Medicine Neonatal networks. https://www.bapm.org/pages/19-neonatal-networks.

[14] British Association of Perinatal Medicine (2022). The british association of perinatal medicine service and quality standards for provision of neonatal care in the uk. https://hubble-live-assets.s3.eu-west-1.amazonaws.com/bapm/file_asset/file/1494/BAPM_Service_Quality_Standards_FINAL.pdf.

[15] Posit team (2024). RStudio: integrated development environment for r. http://www.posit.co.

[16] R Core Team (2021). R: a language and environment for statistical computing. https://www.R-project.org.

[17] Imperial College London Neonatal data analysis unit. https://www.imperial.ac.uk/neonatal-data-analysis-unit/neonatal-data-analysis-unit/.

[18] Simpson DA, Carson C, Kurinczuk JJ (2022). Trends and inequalities in breastfeeding continuation from 1 to 6 weeks: findings from six population-based British cohorts, 1985-2010. Eur J Clin Nutr.

[19] Office for Health Improvement & Disparities (2023). Breastfeeding at 6 to 8 weeks: a comparison of methods. https://www.gov.uk/government/publications/breastfeeding-at-6-to-8-weeks-comparison-of-nhs-england-and-ohid-data/breastfeeding-at-6-to-8-weeks-a-comparison-of-methods#executive-summary.

[20] McFadden A, Renfrew MJ, Atkin K (2013). Does cultural context make a difference to women’s experiences of maternity care? A qualitative study comparing the perspectives of breast-feeding women of Bangladeshi origin and health practitioners. Health Expect.

[21] British Association of Perinatal Medicine Maternal breast milk toolkit. optimising maternal breast milk for preterm infants: a two-part quality improvement toolkit. https://www.bapm.org/pages/196-maternal-breast-milk-toolkit.

[22] Levene I, Quigley MA, Fewtrell M (2024). Does extremely early expression of colostrum after very preterm birth improve mother’s own milk quantity? A cohort study. Arch Dis Child Fetal Neonatal Ed.

[23] Valentine G, Ford S, Hagan J (2021). Percent mother’s own milk feedings for preterm neonates predicts discharge feeding outcomes. J Perinatol.

[24] Dharel D, Singhal N, Wood C (2021). Rates and Determinants of Mother’s Own Milk Feeding in Infants Born Very Preterm. J Pediatr.

[25] Lee HC, Gould JB (2009). Factors influencing breast milk versus formula feeding at discharge for very low birth weight infants in California. J Pediatr.

[26] UNICEF The Baby Friendly Initiative.

[27] Renfrew MJ, Craig D, Dyson L (2009). Breastfeeding promotion for infants in neonatal units: a systematic review and economic analysis. Health Technol Assess.

