# Supplementary material for: Association between maternal sociodemographic characteristics and exclusive mother’s own milk feeding in preterm infants: a cohort study using data from the National Neonatal Research Database
Source: Arch Dis Child Fetal Neonatal Ed. 2025 Feb 26;110(5):e327990. doi: 10.1136/archdischild-2024-327990 (PMC12418531; doi:10.1136/archdischild-2024-327990)
Supplement: online supplemental file 1 [file fetalneonatal-110-5-s001.pdf]

Association between maternal socio-demographic characteristics and exclusive mother's own milk feeding in preterm infants: a cohort study using data from the National Neonatal Research Database.

Online only supplementary material

Supplementary Table 1. Exclusions due to fatal congenital anomalies

Anomaly	Number excluded [a]
Anencephaly and similar malformations	<5
Holoprosencephaly	12
Bilateral renal agenesis/Potter Syndrome	8
Trisomy 13 (Patau's Syndrome) or Trisomy 18 (Edwards' Syndrome)	52
Triploidy/tetraploidy/polyploidy	6
Thanatophoric Dysplasia	<5

[a] Counts <5 suppressed for statistical disclosure control; some infants had >1 anomaly

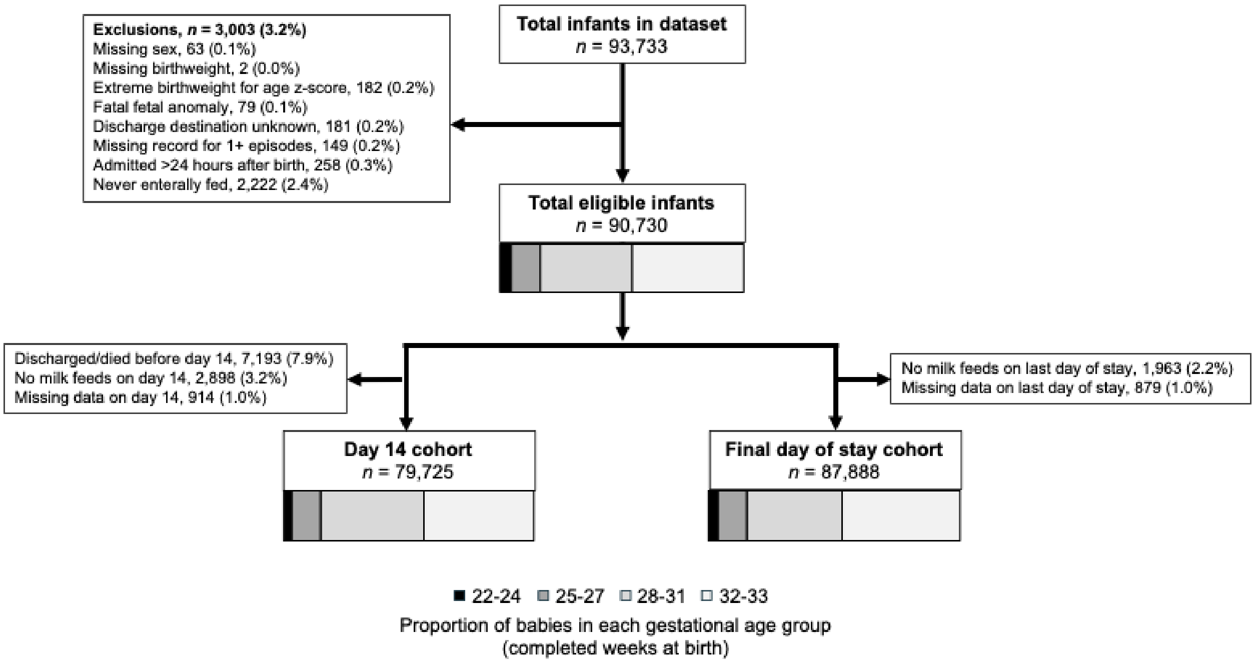

Supplementary Figure 1. Total number of infants included in the analysis.

Bar charts below the boxes denote the proportion of babies in each gestational age subgroup in each analysis.

Supplementary Table 2. Characteristics of infants born at <34 weeks' gestation in England and Wales and admitted for neonatal care (2016 to 2022)

GA at birth category (weeks)	All *n*=90,730	22 to 24 *n*=3,874	25 to 27 *n*=11,160	28 to 31 *n*=34,086	32 to 33 *n*=41,610
**GA at birth (weeks)***	31 (29-33)	24 (23-24)	26 (25-27)	30 (29-31)	33 (32-33)
**Female**	41,357 (45.6)	1,794 (46.3)	5,111 (45.8)	15,294 (44.9)	19,158 (46.0)
**Birth weight (grams)***	1540 (1140-1880)	630 (560-700)	880 (765-1008)	1377 (1160-1590)	1885 (1651-2110)
**Birth weight z-score***	0.00 (-0.60-0.60)	-0.20 (-0.70-0.40)	-0.10 (-0.70-0.50)	0.10 (-0.70-0.60)	0.00 (-0.60-0.60)
**Multiple birth**	23,961 (26.4)	794 (20.5)	2,442 (21.9)	8,961 (26.3)	11,764 (28.3)
**Outcome (discharge or death)**					
**Home inc. foster care**	83,644 (92.2)	2,240 (57.8)	9,449 (84.7)	32,503 (95.4)	39,452 (94.8)
**Discharged to ward**	2,356 (2.6)	132 (3.4)	274 (2.5)	440 (1.3)	1,510 (3.6)
**Transferred out**	1,926 (2.1)	308 (8.0)	520 (4.7)	632 (1.9)	466 (1.1)
**Died**	2,804 (3.1)	1,194 (30.8)	917 (8.2)	511 (1.5)	182 (0.4)
**Length of stay (days)***	33 (20-57)	112 (38-137)	87 (71-106)	45 (34-59)	20 (16-27)
**GA at discharge (weeks)**	36 (35-37)	40 (29-43)	38 (37-41)	36 (35-38)	35 (35-36)
**Born by caesarean section**	54,244 (59.8)	753 (19.4)	5,961 (53.4)	22,293 (65.4)	25,237 (60.7)
**Transferred in first 48 hours**	8,354 (9.2)	716 (18.5)	2,095 (18.8)	3,366 (9.9)	2,177 (5.2)
**NMR-2000 score (risk of in-hospital mortality)**					
**Low risk**	23,621 (26.0)	0 (0.0)	1 (0.0)	6,038 (17.7)	17,582 (42.3)
**Medium risk**	40,408 (44.5)	1,334 (34.4)	8,634 (77.4)	23,736 (69.6)	6,704 (16.1)
**High risk**	3,970 (4.4)	2,147 (55.4)	1,418 (12.7)	397 (1.2)	8 (0.0)
**NA, BW <2000g**	15,673 (17.3)	0 (0.0)	0 (0.0)	792 (2.3)	14,881 (35.8)
**Missing**	7,058 (7.8)	393 (10.1)	1,107 (9.9)	3,123 (9.2)	2,435 (5.9)
**Maternal age (years)**					
**<16**	54 (0.1)	2 (0.1)	14 (0.1)	17 (0.0)	21 (0.1)
**16-25**	17,237 (19.0)	745 (19.2)	2,232 (20.0)	6,593 (19.3)	7,667 (18.4)
**26-35**	50,899 (56.1)	2,174 (56.1)	6,334 (56.8)	19,073 (56.0)	23,318 (56.0)
**36-45**	20,820 (22.9)	887 (22.9)	2,434 (21.8)	7,761 (22.8)	9,738 (23.4)
**46+**	914 (1.0)	42 (1.1)	85 (0.8)	357 (1.0)	430 (1.0)
**Missing**	806 (0.9)	24 (0.6)	61 (0.5)	285 (0.8)	436 (1.0)
**Maternal ethnic group**					
**White**	52,608 (58.0)	1,866 (48.2)	5,976 (53.5)	19,858 (58.3)	24,908 (59.9)
**Mixed**	1,514 (1.7)	72 (1.9)	193 (1.7)	566 (1.7)	683 (1.6)
**Asian/Asian British**	9,629 (10.6)	515 (13.3)	1,305 (11.7)	3,639 (10.7)	4,170 (10.0)
**Black/Black British**	5,621 (6.2)	457 (11.8)	977 (8.8)	2,140 (6.3)	2,047 (4.9)
**Other**	1,783 (2.0)	87 (2.2)	253 (2.3)	663 (1.9)	780 (1.9)
**Index of Multiple Deprivation quintile (from maternal postcode)**					
**Most deprived**	25,906 (28.6)	1,256 (32.4)	3,385 (30.3)	9,839 (28.9)	11,426 (27.5)

**2**	19,353 (21.3)	873 (22.5)	2,498 (22.4)	7,223 (21.2)	8,759 (21.1)
**3**	15,566 (17.2)	638 (16.5)	1,941 (17.4)	5,820 (17.1)	7,167 (17.2)
**4**	12,982 (14.3)	515 (13.3)	1,467 (13.1)	4,926 (14.5)	6,074 (14.6)
**Least deprived**	10,674 (11.8)	352 (9.1)	1,177 (10.5)	3,941 (11.6)	5,204 (12.5)
**Missing**	6,249 (6.9)	240 (6.2)	692 (6.2)	2,337 (6.9)	2,980 (7.2)
**Level of neonatal care**					
**Level 3 (Neonatal Intensive Care Unit)**	46,786 (51.6)	3,565 (92.0)	9,333 (83.6)	17,456 (51.2)	16,432 (39.5)
**Level 2 (Local Neonatal Unit)**	34,674 (38.2)	82 (2.1)	1,250 (11.2)	14,132 (41.5)	19,210 (46.2)
**Level 3 (Special Care Baby Unit)**	6,699 (7.4)	106 (2.7)	236 (2.1)	1,511 (4.4)	4,846 (11.7)
**Missing**	2,571 (2.8)	121 (3.1)	341 (3.1)	987 (2.9)	1,122 (2.7)
**Neonatal Operational Delivery Network (ODN) of care received**					
**ODN 1**	10,338 (11.4)	433 (11.2)	1,249 (11.2)	3,904 (11.5)	4,752 (11.4)
**ODN 2**	9,177 (10.1)	293 (7.6)	1,035 (9.3)	3,577 (10.5)	4,272 (10.3)
**ODN 3**	8,977 (9.9)	440 (11.4)	1,289 (11.6)	3,399 (10.0)	3,849 (9.3)
**ODN 4**	8,271 (9.1)	263 (6.8)	825 (7.4)	3,066 (9.0)	4,117 (9.9)
**ODN 5**	7,337 (8.1)	354 (9.1)	874 (7.8)	2,727 (8.0)	3,382 (8.1)
**ODN 6**	7,317 (8.1)	306 (7.9)	972 (8.7)	2,837 (8.3)	3,202 (7.7)
**ODN 7**	6,385 (7.0)	299 (7.7)	823 (7.4)	2,298 (6.7)	2,965 (7.1)
**ODN 8**	6,224 (6.9)	195 (5.0)	630 (5.6)	2,363 (6.9)	3,036 (7.3)
**ODN 9**	5,973 (6.6)	348 (9.0)	796 (7.1)	2,100 (6.2)	2,729 (6.6)
**ODN 10**	5,931 (6.5)	232 (6.0)	629 (5.6)	2,257 (6.6)	2,813 (6.8)
**ODN 11**	5,419 (6.0)	263 (6.8)	777 (7.0)	2,066 (6.1)	2,313 (5.6)
**ODN 12**	3,753 (4.1)	254 (6.6)	660 (5.9)	1,387 (4.1)	1,452 (3.5)
**ODN 13**	3,057 (3.4)	73 (1.9)	260 (2.3)	1,118 (3.3)	1,606 (3.9)
**Missing**	2,571 (2.8)	121 (3.1)	341 (3.0)	987 (2.9)	1,122 (2.7)

Figures are *n* (%) except those marked * which are median (interquartile range)
GA, gestational age; BW, birth weight; ODN, Operational Delivery Network

Supplementary Table 3. Milk received during neonatal care, by gestational age group

Milk received throughout neonatal care, *n* (%)					
**GA at birth category (weeks)**	**All *n*=90,730**	**22 to 24 *n*=3,874**	**25 to 27 *n*=11,160**	**28 to 31 *n*=34,086**	**32 to 33 *n*=41,610**
**Exclusively MOM**	11,962 (13.2)	898 (23.2)	1,884 (16.9)	5,182 (15.2)	3,998 (9.6)
**No MOM**	9,018 (9.9)	78 (2.0)	193 (1.7)	1,937 (5.7)	6,810 (16.4)
**Exclusively human milk**	17,261 (19.0)	1,363 (35.2)	2,855 (25.6)	7,725 (22.7)	5,318 (12.8)
**No human milk**	8,033 (8.9)	15 (0.4)	83 (0.7)	1,465 (4.3)	6,470 (15.5)
**Any MOM**	80,884 (89.1)	3,629 (93.7)	10,786 (96.6)	31,800 (93.3)	34,669 (83.3)
**Any DHM**	23,785 (26.2)	1,777 (45.9)	5,199 (46.6)	11,836 (34.7)	4,973 (12.0)
**Any formula**	73,168 (80.6)	2,365 (61.0)	8,230 (73.7)	26,318 (77.2)	36,255 (87.1)
**First day of life of receiving type of milk, median (1QR)**					
**MOM**	3 (2-4)	4 (2-5)	3 (2-5)	3 (2-4)	2 (2-4)
**DHM**	3 (2-6)	4 (2-19)	3 (2-12)	3 (2-5)	2 (2-4)
**Formula**	4 (2-21)	62 (38-89)	40 (23-60)	12 (3-26)	2 (1-4)
**Received any MOM on day 1 or 2, *n* (%)**	36,021 (39.7)	1,021 (26.4)	3,487 (31.2)	13,416 (39.4)	18,097 (43.5)
**Milk fed on day 14, *n* (%)**					
**GA at birth category (weeks)**	**All *n*=79,725**	**22 to 24 *n*=2,455**	**25 to 27 *n*=9,571**	**28 to 31 *n*=32,625**	**32 to 33 *n*=35,074**
**Exclusively MOM**	43,869 (55.0)	2,022 (82.4)	7,424 (77.6)	19,262 (59.0)	15,161 (43.2)
**No MOM**	14,738 (18.5)	142 (5.8)	736 (7.7)	4,718 (14.5)	9,142 (26.1)
**Exclusively human milk**	50,039 (62.8)	2,388 (97.3)	8,934 (93.3)	22,831 (70.0)	15,886 (45.3)
**No human milk**	12,443 (15.6)	19 (0.8)	188 (2.0)	3,430 (10.5)	8,806 (25.1)
**Any MOM**	64,987 (81.5)	2,313 (94.2)	8,835 (92.3)	27,907 (85.5)	25,932 (73.9)
**Any DHM**	7,892 (9.9)	375 (15.3)	1,619 (16.9)	4,631 (14.2)	1,267 (3.6)
**Any formula**	17,243 (21.6)	48 (2.0)	449 (4.7)	6,363 (19.5)	10,382 (29.6)
**Milk fed on last day of neonatal care, *n* (%)**					
**GA at birth category (weeks)**	**All *n*=87,888**	**22 to 24 *n*=2,986**	**25 to 27 *n*=10,365**	**28 to 31 *n*=33,382**	**32 to 33 *n*=41,155**
**Exclusively MOM**	31,450 (34.7)	886 (22.9)	3,178 (28.5)	12,027 (35.5)	15,359 (36.9)
**No MOM**	3,4941 (38.5)	1425 (36.8)	4911 (44.0)	13567 (39.8)	15038 (36.1)
**Exclusively human milk**	31,677 (34.9)	947 (24.4)	3,228 (28.9)	12,084 (35.5)	15,418 (37.1)
**No human milk**	34,816 (38.4)	1,398 (36.1)	4,874 (43.7)	13,542 (39.7)	15,002 (36.1)
**Any MOM**	52,947 (58.4)	1,561 (40.3)	5,454 (48.9)	19,815 (58.1)	26,117 (62.8)
**Any DHM**	327 (0.4)	65 (1.7)	61 (0.5)	96 (0.3)	105 (0.3)
**Any formula**	56,211 (62.0)	2,039 (52.6)	7,137 (64.0)	21,298 (62.5)	25,737 (61.9)
DHM, donor human milk; GA, gestational age; IQR, interquartile range; MOM, their mother's own milk					

Supplementary Table 4. Adjusted and unadjusted odds ratios for exclusive and no MOM, by maternal socio-demographic characteristics

		Exclusive MOM throughout stay			No MOM ever during stay		
		Unadjusted	Mutually adjusted + clustered	Fully adjusted + clustered	Unadjusted	Mutually adjusted + clustered	Fully adjusted + clustered
IMD quintile	Most deprived	Ref	Ref	Ref	Ref	Ref	Ref
	2	1.17 (1.11-1.24)	1.34 (1.26-1.43)	1.37 (1.29-1.46)	0.63 (0.60-0.67)	0.69 (0.65-0.74)	0.68 (0.64-0.73)
	3	1.29 (1.22-1.37)	1.55 (1.46-1.66)	1.62 (1.51-1.73)	0.52 (0.49-0.56)	0.55 (0.51-0.59)	0.54 (0.50-0.58)
	4	1.45 (1.36-1.54)	1.73 (1.62-1.85)	1.85 (1.72-1.98)	0.47 (0.44-0.51)	0.47 (0.43-0.51)	0.44 (0.41-0.48)
	Least deprived	1.57 (1.47-1.67)	1.99 (1.85-2.14)	2.16 (2.01-2.33)	0.36 (0.33-0.40)	0.36 (0.32-0.39)	0.33 (0.30-0.36)
	Missing	1.22 (1.13-1.33)	1.37 (1.21-1.56)	1.44 (1.26-1.64)	0.70 (0.64-0.76)	0.77 (0.67-0.88)	0.72 (0.62-0.82)
Maternal ethnic group	White	Ref	Ref	Ref	Ref	Ref	Ref
	Mixed	1.12 (0.97-1.30)	1.16 (1.00-1.35)	1.16 (1.00-1.35)	0.58 (0.48-0.71)	0.63 (0.52-0.78)	0.63 (0.51-0.78)
	Asian/Asian British	0.86 (0.81-0.92)	0.94 (0.88-1.01)	0.88 (0.82-0.95)	0.33 (0.29-0.36)	0.36 (0.32-0.40)	0.37 (0.33-0.42)
	Black/Black British	1.09 (1.01-1.18)	1.19 (1.09-1.29)	1.07 (0.98-1.17)	0.34 (0.30-0.39)	0.39 (0.34-0.44)	0.44 (0.38-0.50)
	Other	1.26 (1.11-1.43)	1.28 (1.12-1.46)	1.23 (1.07-1.41)	0.30 (0.23-0.38)	0.34 (0.27-0.44)	0.34 (0.26-0.43)
	Missing	0.96 (0.92-1.01)	0.99 (0.94-1.05)	0.96 (0.91-1.02)	0.89 (0.85-0.94)	0.91 (0.86-0.96)	0.93 (0.87-0.99)
Maternal age group	<26	0.65 (0.62-0.69)	0.68 (0.64-0.72)	0.64 (0.61-0.68)	1.41 (1.34-1.49)	1.13 (1.07-1.20)	1.18 (1.11-1.25)
	26-35	Ref	Ref	Ref	Ref	Ref	Ref
	36-45	0.88 (0.84-0.93)	0.87 (0.83-0.91)	0.91 (0.86-0.95)	0.95 (0.90-1.01)	1.12 (1.06-1.19)	1.14 (1.07-1.21)
	46+	0.38 (0.29-0.50)	0.38 (0.29-0.50)	0.50 (0.37-0.66)	0.65 (0.50-0.85)	1.02 (0.78-1.34)	1.06 (0.80-1.40)
	Missing	0.84 (0.68-1.03)	0.88 (0.70-1.09)	0.90 (0.72-1.12)	1.41 (1.15-1.74)	1.26 (1.01-1.56)	1.14 (0.91-1.42)
Gestational age (weeks)	33			Ref			Ref
	32			1.46 (1.36-1.57)			0.83 (0.78-0.88)
	31			1.93 (1.77-2.11)			0.66 (0.60-0.72)
	30			2.28 (2.07-2.52)			0.58 (0.52-0.66)
	29			3.40 (3.05-3.80)			0.42 (0.36-0.49)
	28			4.33 (3.85-4.87)			0.30 (0.25-0.37)
	27			5.26 (4.62-5.99)			0.27 (0.22-0.35)
	26			5.61 (4.90-6.42)			0.17 (0.13-0.23)
	25			5.12 (4.43-5.92)			0.13 (0.09-0.20)
	24			5.32 (4.57-6.21)			0.19 (0.13-0.26)
	23			6.78 (5.64-8.15)			0.20 (0.12-0.31)
	22			7.39 (5.21-10.49)			0.39 (0.19-0.77)
Sex	Male			Ref			Ref
	Female			0.91 (0.87-0.94)			1.05 (1.01-1.10)
Multiple birth	Singleton			Ref			Ref
	Multiple			0.55 (0.52-0.57)			1.05 (0.99-1.11)
Mode of delivery	Vaginal			Ref			Ref
	Caesarean			0.95 (0.91-1.00)			0.89 (0.85-0.94)
	Missing			0.88 (0.80-0.97)			0.98 (0.89-1.08)
Acute post-natal transfer	No			Ref			Ref
	Yes			0.94 (0.87-1.00)			1.06 (0.97-1.16)
NMR-2000 risk of in hospital mortality	Low risk			Ref			Ref
	Medium risk			1.40 (1.31-1.50)			0.83 (0.77-0.89)
	High risk			1.48 (1.30-1.68)			0.71 (0.52-0.96)
	BW >2000g			0.97 (0.90-1.04)			1.07 (1.01-1.14)
	Missing			1.20 (1.09-1.32)			0.98 (0.88-1.08)
Length of stay (weeks)	>8			Ref			Ref
	6-8			2.65 (2.45-2.87)			1.19 (1.04-1.37)
	4-6			3.45 (3.17-3.74)			1.33 (1.17-1.52)
	2-4			3.91 (3.57-4.27)			2.18 (1.91-2.48)
	≤2			5.35 (4.83-5.92)			3.77 (3.27-4.34)
Unit level	Level 3 (NICU)			Ref			Ref
	Level 2 (LNU)			0.75 (0.61-0.92)			1.03 (0.90-1.18)
	Level 1 (SCBU)			0.55 (0.42-0.71)			0.96 (0.82-1.14)
	Missing			0.81 (0.44-1.49)			0.91 (0.45-1.84)
LR test chi^2 vs logistic model (*p* value)			2620.440 (<0.001)	2376.081 (<0.001)		1509.273 (<0.001)	829.085 (<0.001)
Variance (SE) of random intercept for ODN			0.045 (0.031)	0.060 (0.036)		0.113 (0.051)	0.103 (0.044)
Variance (SE) of random intercept for Unit			0.358 (0.047)	0.300 (0.040)		0.211 (0.028)	0.107 (0.016)

Supplementary Figure 2. Unadjusted and adjusted* odds ratios (95% confidence intervals) for exclusively receiving human milk (mother's own milk and/or donor human milk) and not receiving any human milk throughout neonatal care.

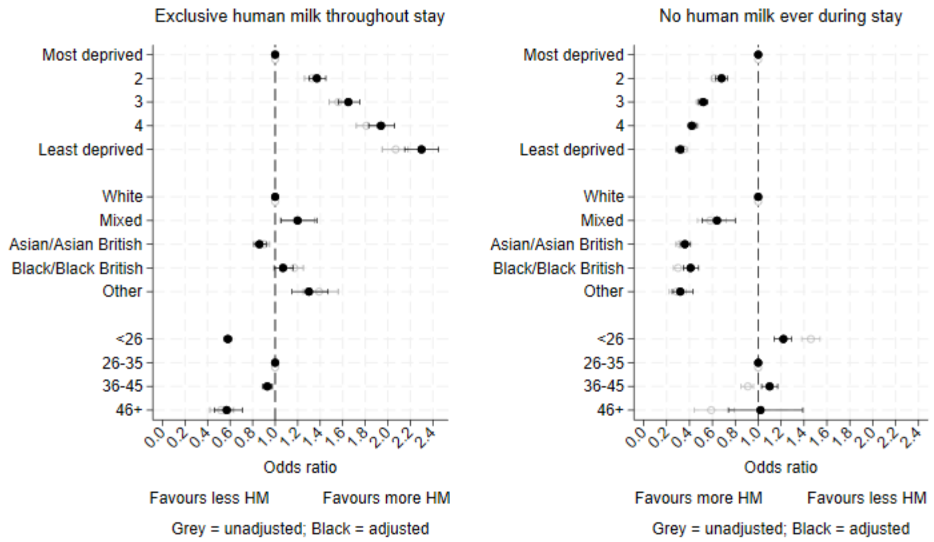

*Adjusted for other variables shown, plus the following: GA in completed weeks; sex; multiple birth; mode of delivery; NMR-2000 score indicating risk of in-hospital mortality; inter-hospital transfer in first 48 hours of life; length of hospital stay (in two week bands); unit level. To account for clustering, adjusted models included random effects for ODN and unit.

Supplementary Table 5. Adjusted and unadjusted odds ratios for exclusive and human milk, and no human milk by maternal socio-demographic characteristics

		Exclusive MOM throughout stay			No MOM ever during stay		
		Unadjusted	Mutually adjusted + clustered	Fully adjusted + clustered	Unadjusted	Mutually adjusted + clustered	Fully adjusted + clustered
IMD quintile	Most deprived	Ref	Ref	Ref	Ref	Ref	Ref
	2	1.33 (1.26-1.39)	1.33 (1.26-1.41)	1.37 (1.30-1.45)	0.62 (0.59-0.66)	0.70 (0.66-0.75)	0.68 (0.63-0.73)
	3	1.56 (1.48-1.65)	1.56 (1.48-1.66)	1.65 (1.56-1.75)	0.49 (0.46-0.53)	0.54 (0.50-0.58)	0.52 (0.48-0.56)
	4	1.81 (1.72-1.91)	1.79 (1.68-1.89)	1.94 (1.83-2.06)	0.44 (0.41-0.48)	0.46 (0.42-0.50)	0.42 (0.39-0.46)
	Least deprived	2.07 (1.95-2.18)	2.06 (1.93-2.19)	2.30 (2.15-2.45)	0.35 (0.32-0.38)	0.35 (0.32-0.39)	0.32 (0.28-0.35)
	Missing	1.53 (1.43-1.65)	1.36 (1.22-1.52)	1.44 (1.28-1.61)	0.68 (0.62-0.75)	0.78 (0.67-0.90)	0.70 (0.60-0.82)
Maternal ethnic group	White	Ref	Ref	Ref	Ref	Ref	Ref
	Mixed	1.19 (1.05-1.35)	1.20 (1.05-1.36)	1.20 (1.05-1.37)	0.58 (0.47-0.72)	0.64 (0.52-0.79)	0.64 (0.51-0.80)
	Asian/Asian British	0.90 (0.85-0.95)	0.93 (0.88-0.99)	0.86 (0.81-0.92)	0.32 (0.28-0.35)	0.35 (0.31-0.39)	0.36 (0.32-0.41)
	Black/Black British	1.17 (1.09-1.25)	1.21 (1.12-1.30)	1.07 (0.99-1.16)	0.30 (0.26-0.35)	0.36 (0.31-0.42)	0.41 (0.35-0.48)
	Other	1.39 (1.25-1.56)	1.35 (1.20-1.52)	1.30 (1.15-1.47)	0.29 (0.22-0.37)	0.33 (0.25-0.43)	0.32 (0.25-0.43)
	Missing	1.03 (0.99-1.08)	1.02 (0.97-1.06)	0.98 (0.93-1.03)	0.88 (0.83-0.93)	0.90 (0.85-0.96)	0.93 (0.87-0.99)
Maternal age group	<26	0.58 (0.55-0.60)	0.62 (0.59-0.65)	0.58 (0.55-0.61)	1.46 (1.38-1.54)	1.16 (1.09-1.23)	1.22 (1.14-1.29)
	26-35	Ref	Ref	Ref	Ref	Ref	Ref
	36-45	0.94 (0.90-0.98)	0.89 (0.85-0.92)	0.93 (0.89-0.97)	0.91 (0.85-0.96)	1.08 (1.02-1.15)	1.10 (1.03-1.17)
	46+	0.52 (0.42-0.63)	0.45 (0.37-0.55)	0.57 (0.46-0.71)	0.59 (0.44-0.79)	0.98 (0.72-1.33)	1.02 (0.74-1.39)
	Missing	0.94 (0.79-1.12)	0.99 (0.83-1.19)	1.02 (0.84-1.23)	1.43 (1.15-1.78)	1.26 (1.00-1.58)	1.14 (0.90-1.45)
Gestational age (weeks)	33			Ref			Ref
	32			1.58 (1.48-1.68)			0.79 (0.74-0.84)
	31			2.30 (2.13-2.48)			0.61 (0.55-0.67)
	30			2.87 (2.63-3.13)			0.48 (0.42-0.55)
	29			4.45 (4.03-4.90)			0.27 (0.22-0.33)
	28			6.27 (5.64-6.97)			0.19 (0.15-0.24)
	27			7.68 (6.84-8.63)			0.15 (0.11-0.21)
	26			8.16 (7.23-9.21)			0.08 (0.05-0.12)
	25			7.73 (6.79-8.80)			0.08 (0.05-0.13)
	24			8.43 (7.34-9.67)			0.07 (0.04-0.13)
	23			11.35 (9.58-13.46)			0.03 (0.01-0.11)
	22			16.51 (11.69-23.32)			0.06 (0.01-0.43)
Sex	Male			Ref			Ref
	Female			0.94 (0.91-0.97)			1.08 (1.02-1.13)
Multiple birth	Singleton			Ref			Ref
	Multiple			0.57 (0.55-0.60)			1.06 (1.00-1.13)
Mode of delivery	Vaginal			Ref			Ref
	Caesarean			1.00 (0.96-1.04)			0.84 (0.80-0.89)
	Missing			0.93 (0.85-1.01)			0.94 (0.85-1.04)
Acute post-natal transfer	No			Ref			Ref
	Yes			0.90 (0.85-0.96)			1.01 (0.91-1.12)
NMR-2000 risk of in hospital mortality	Low risk			Ref			Ref
	Medium risk			1.42 (1.34-1.50)			0.74 (0.68-0.80)
	High risk			1.35 (1.21-1.51)			0.38 (0.21-0.68)
	BW >2000g			0.88 (0.82-0.94)			1.10 (1.03-1.17)
	Missing			1.28 (1.18-1.39)			0.94 (0.84-1.05)
Length of stay (weeks)	>8			Ref			Ref
	6-8			3.14 (2.93-3.37)			1.13 (0.96-1.34)
	4-6			4.49 (4.17-4.84)			1.36 (1.16-1.60)
	2-4			4.94 (4.55-5.36)			2.26 (1.92-2.65)
	≤2			6.98 (6.34-7.68)			3.79 (3.20-4.49)
Unit level	Level 3 (NICU)			Ref			Ref
	Level 2 (LNU)			0.72 (0.60-0.86)			1.04 (0.89-1.22)
	Level 1 (SCBU)			0.46 (0.37-0.57)			1.01 (0.84-1.21)
	Missing			0.80 (0.45-1.43)			0.88 (0.38-2.02)
LR test chi^2 vs logistic model (*p* value)			2936.650 (<0.001)	2126.002 (<0.001)		1877.931 (<0.001)	1051.251 (<0.001)
Variance (SE) of random intercept for ODN			0.047 (0.029)	0.059 (0.031)		0.155 (0.072)	0.147 (0.063)
Variance (SE) of random intercept for Unit			0.348 (0.045)	0.223 (0.030)		0.305 (0.040)	0.150 (0.023)

1. Only human milk throughout neonatal care

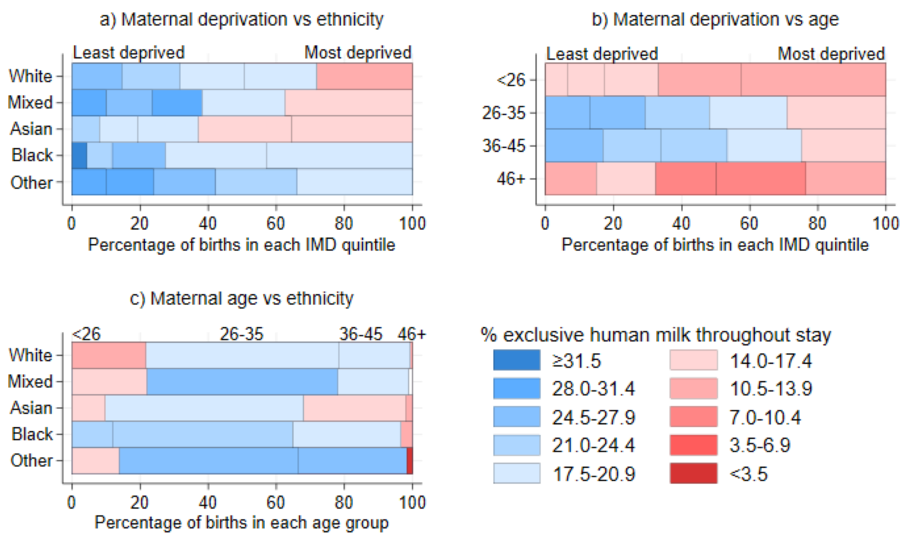

2. No human milk throughout neonatal care

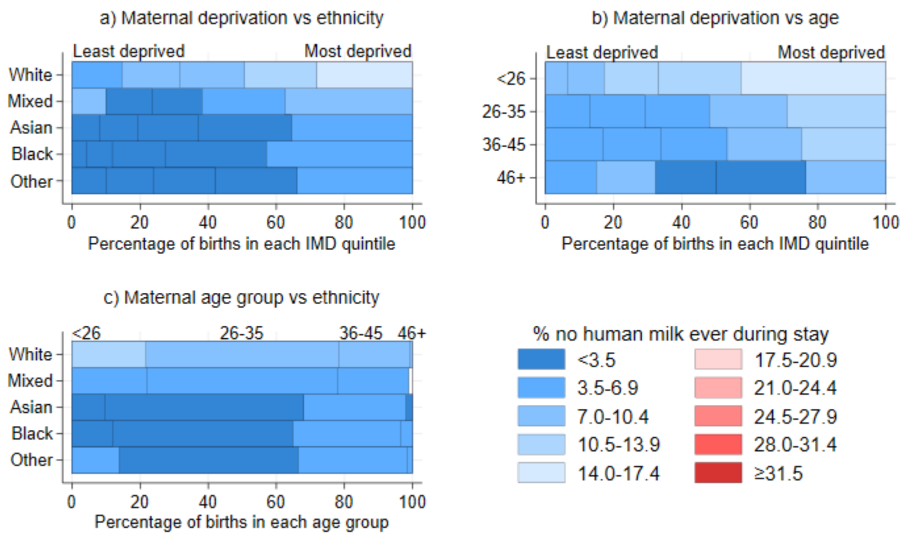

Supplementary Figure 3. Exclusive human milk feeding (1) and no human milk feeding (2) throughout neonatal care by maternal Index of Multiple Deprivation (IMD) quintile and ethnicity (a), IMD and age (b), and age and ethnicity (c). Data suppressed for groups with <20 infants.

1. Only mother's own milk throughout neonatal care

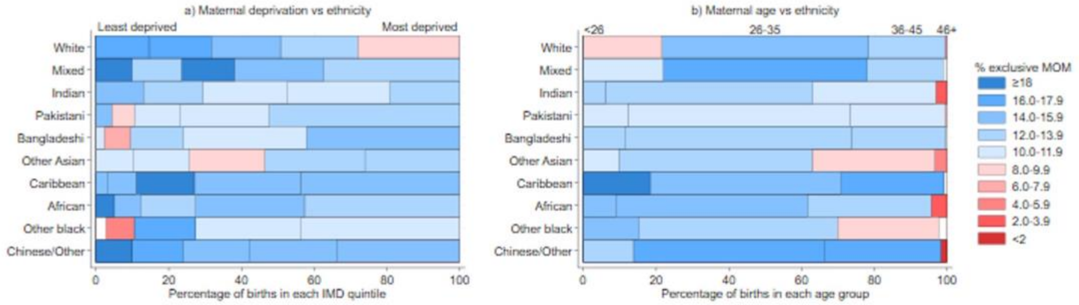

2. No mother's own milk throughout neonatal care

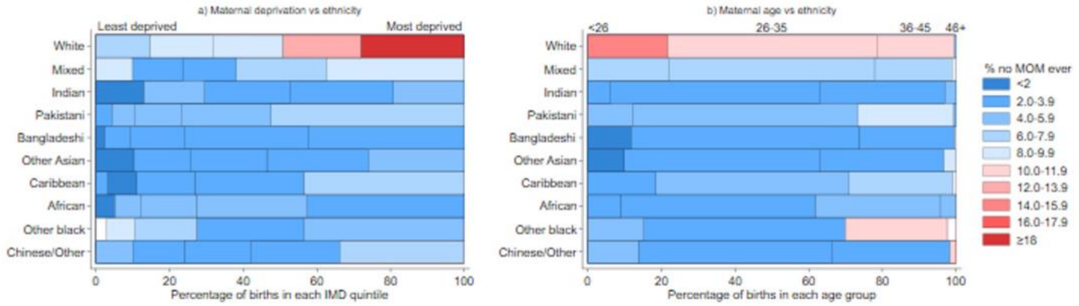

1. Only human milk throughout neonatal care

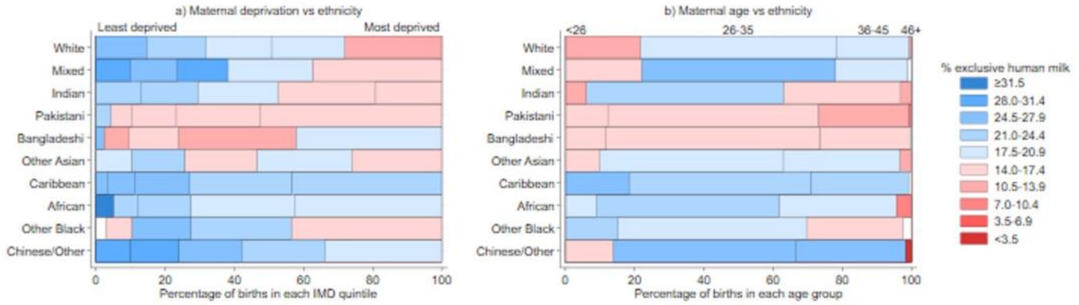

2. No human milk throughout neonatal care

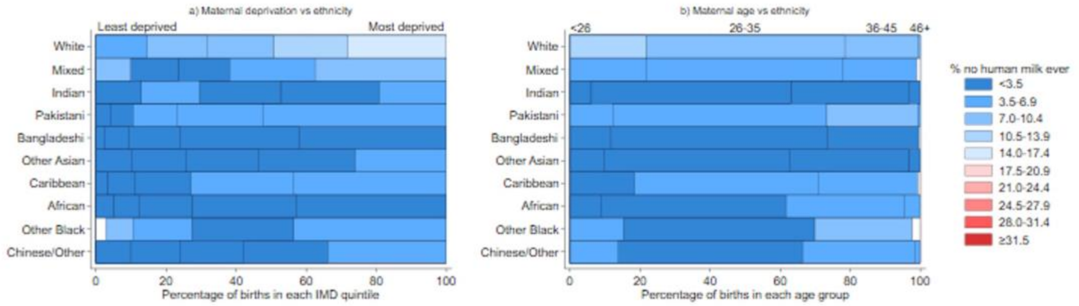

Supplementary Figure 4. Variations in mother's own milk and human milk feeding by dis-aggregated ethnic group (data suppressed for groups with <20 infants)

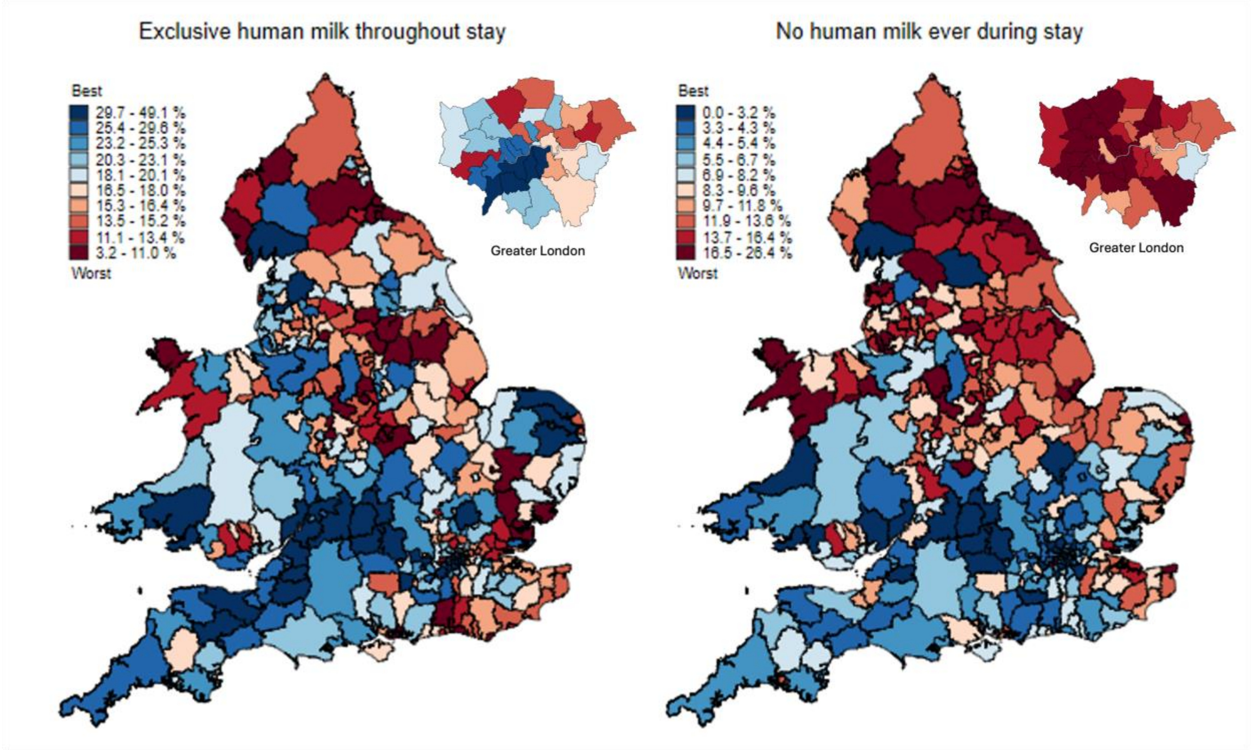

Supplementary Figure 5. Percentage of infants by maternal Local Authority District (LAD) who exclusively received human milk (their mother's own milk and or human donor milk) and did not receive any human milk throughout neonatal care in <34 weeks' gestational age infants admitted for neonatal care in England and Wales (2016 to 2022).
